# Rapidly Progressive Myelopathy Caused by Aggressive Vertebral Hemangioma

**DOI:** 10.1155/2019/8927310

**Published:** 2019-11-15

**Authors:** Kevin A. Moattari, Rojeh Melikian, Sanjay K. Khurana

**Affiliations:** DISC Sports & Spine Center, 13160 Mindanao Way, Suite 300, Marina del Rey, CA 90292, USA

## Abstract

**Introduction:**

Vertebral hemangiomas are the most common benign tumors of the spine, having an incidence of 10-12% in the general population. They are asymptomatic, incidental findings in the vast majority of patients; however, in rare cases, they can expand to cause neural compression. Aggressive lesions of this sort are most commonly found in the thoracic spine, and expansion leads to the subacute development of myelopathy.

**Case Report:**

The authors report a rare case of aggressive vertebral hemangioma at the T1 vertebral body which caused rapidly progressive myelopathy over the course of 7 days. Clinical and radiological findings are shown as well as surgical management of the lesion. The patient regained the ability to ambulate, and there was no evidence of disease recurrence at 2-year follow-up.

**Conclusions:**

Although aggressive vertebral hemangiomas are a rare cause of myelopathy, they must be kept in mind in the differential diagnosis of cord compressive lesions. In this case, contrary to most, the expansion of the hemangioma led to rapid development of neurological decline necessitating urgent surgical intervention.

## 1. Introduction

Vertebral hemangiomas (VHs) are the most common benign tumors of the spine [1]. They are developmental neoplasms of endothelial cells that grow within the marrow of the vertebral body [2]. VHs have an incidence of 10-12% in the general population based on postmortem studies and MRI reviews [3–6]. In the vast majority of patients, they remain asymptomatic and do not require any treatment. Asymptomatic lesions are often labeled as incidental findings once discovered on imaging studies. However, in 0.9-1.2% of patients, VHs can expand to cause pain and neural compression [7–9]. In this circumstance, the VH is termed aggressive. Aggressive VHs can cause neural compression via numerous mechanisms, including epidural extension of the soft tissue component of the tumor tissue, bony element expansion, compression from large feeding vessels as a result of angiogenesis, epidural hematoma, or spinal instability caused by vertebral compression fracture [3, 4, 10, 11]. Aggressive VHs are more common in adults and may be more prone to present in female patients during the last trimester of pregnancy [12, 13]. Multilevel hemangiomas are rare but have been reported. A multifocal pain pattern and significant change in pain characteristics are indicators to thoroughly investigate for multiple-level hemangiomas which may have been overlooked on plain radiography [14].

Treatment options for aggressive VHs include radiation therapy, endovascular or percutaneous embolization, vertebroplasty, ethanol injection, or surgical intervention [15]. The latter is generally warranted for neurological compromise or for pain refractory to other measures. This case report illustrates an aggressive vertebral hemangioma causing rapidly progressive myelopathy due to extraosseous, epidural extension of tissue, and cord compression. This is atypical as compared to the insidious onset of symptoms in most cases of aggressive hemangiomas. The location of the aggressive T1 VH is also uncommon as most tend to be found between T3 and T9 [10]. Nevertheless, as this case demonstrates, aggressive VG must be kept in mind for the differential diagnosis for thoracic lesions causing cord compression.

## 2. Presentation of the Case

**Figure 1:**
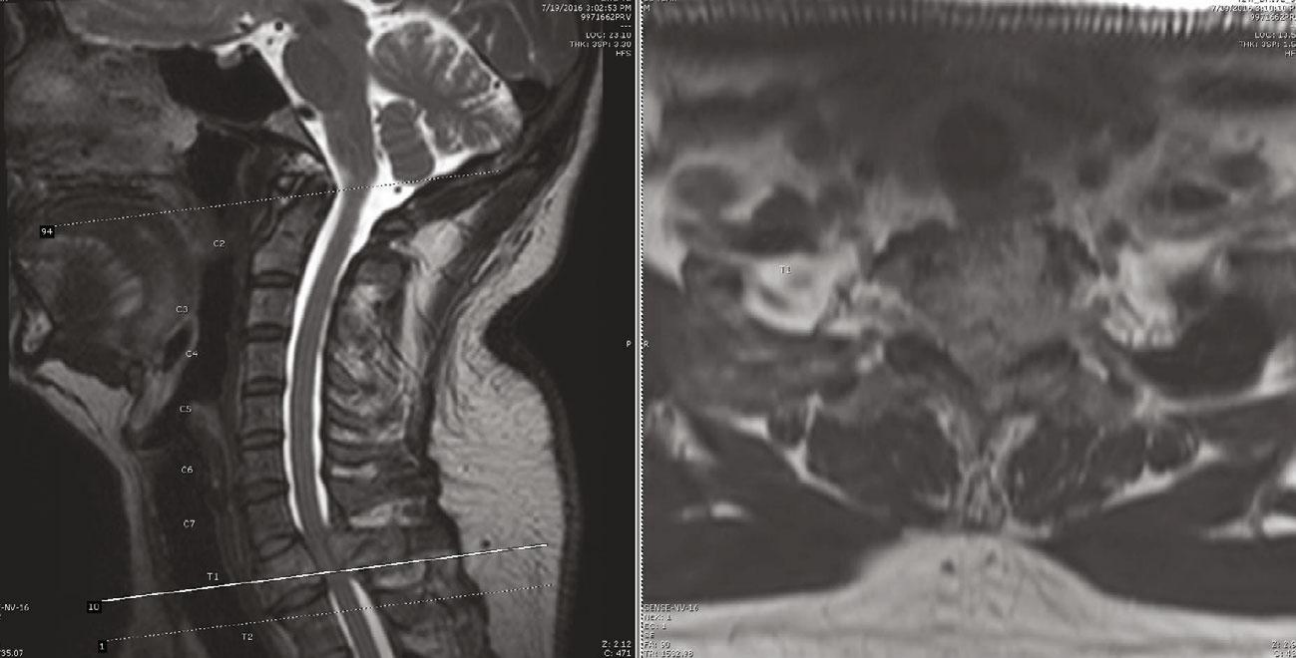
Preoperative T2 magnetic resonance imaging showing sagittal and axial views.

**Figure 2:**
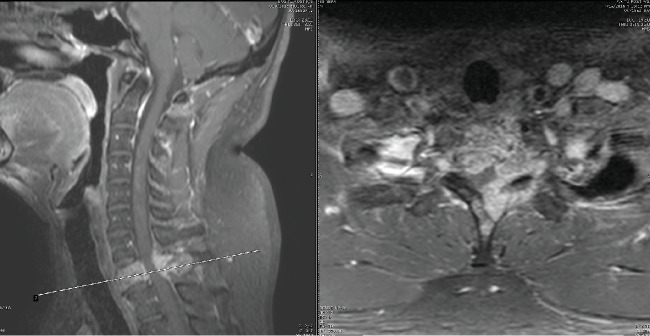
Preoperative T1 magnetic resonance imaging showing sagittal and axial views.

**Figure 3:**
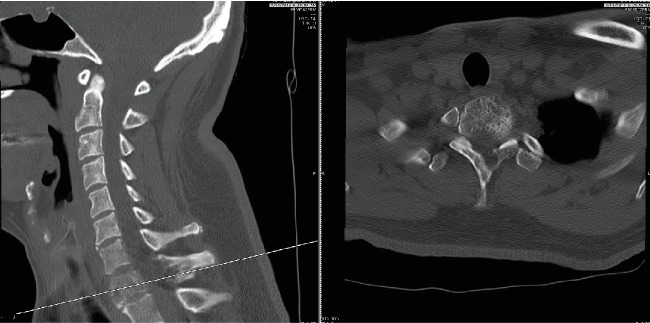
Preoperative computed tomography scan showing sagittal and axial views.

Our patient is an otherwise healthy 50-year-old male who presented to the clinic with complaint of severe gait disturbance progressing rapidly over the past week. MRI done in an outpatient setting demonstrated a lesion at T1 with epidural extension and cord compression. He was sent to the emergency department for further workup. He had neither history of smoking nor any risk factors for malignancy. He had no unintended weight loss, night sweats, or fevers. On physical exam, he had full strength in all upper and lower extremity muscle groups but demonstrated decreased sensation globally from his T3 dermatome down. He had 4 beats of clonus bilaterally and was hyperreflexic in his lower extremities. He had profound ataxia and was unable to ambulate more than a few steps. Contrasted MRI of his thoracic spine demonstrated a diffusely enhancing lesion at T1 with near circumferential epidural disease causing severe cord compression (Figures 1 and 2). CT scan of the thoracic spine demonstrated an expansile bony lesion at the T1 body extending posteriorly into the pedicle and posterior elements on the left side (Figure 3). The chest and abdomen/pelvis CT scans were negative for any evidence of a primary lesion, and all lab workup returned to be normal.

Given the patient's rapidly progressive neurological decline, it was decided to proceed urgently to the operating room to allow for decompression and stabilization of his spinal cord and to obtain a tissue diagnosis. The patient underwent a C6-T3 posterior instrumentation and fusion along with T1 laminectomy and partial C7 and T2 laminectomies. This accomplished the goals for spinal cord decompression and stabilization. Blood loss at this point approached 600 mL given the bleeding from the tumor itself. In the frozen section, we were unable to identify what the lesion was, and so it was decided to hold off on the anterior corpectomy until we had a more definitive diagnosis. A few days later, the permanent pathology returned with a diagnosis of aggressive VH.

**Figure 4:**
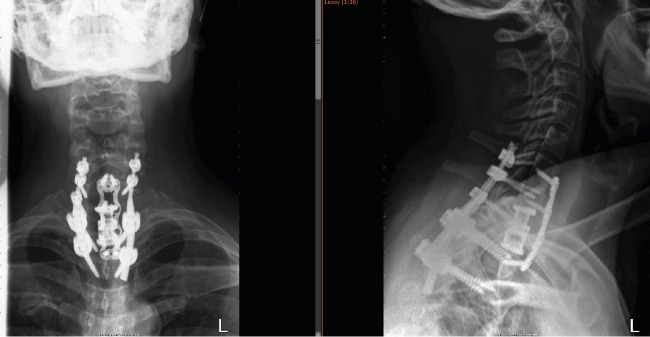
Postoperative AP and lateral X-ray after the second stage of surgery.

**Figure 5:**
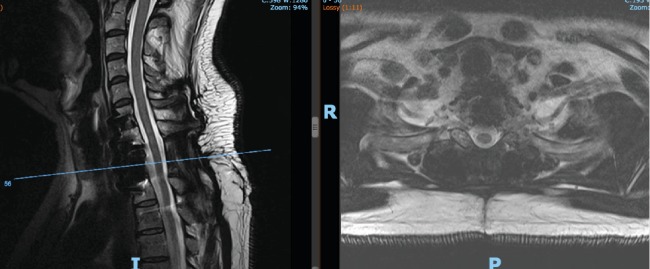
T2 magnetic resonance imaging at two-year follow-up appointment showing sagittal and axial views.

Given the bleeding encountered during the posterior procedure, we opted to have the patient undergo preoperative embolization prior to the planned corpectomy. Angiography at the time of embolization demonstrated a medial branch off the left thyrocervical trunk which supplied numerous vascular lakes inside the T1 vertebral body. Embolization was then carried out with Trufill mixed in a ratio of 1 : 3 with estradiol oil. Postembolization angiography showed no further filling of the hemangioma. The day after embolization, the patient was returned to the operating room for the anterior approach for T1 corpectomy and C7-T2 cage placement/anterior plating (Figure 4). Postoperatively, the patient did well and was discharged home a few days thereafter. His gait rapidly improved postoperatively and continued to improve to close to normal at 1 year postoperatively. Given the location of his lesion and the high likelihood of residual disease, we opted for radiation treatment to limit the growth of any residual disease. On postoperative MRI at 2-year follow-up, there has been no recurrence of the disease (Figure 5).

## 3. Discussion

**Figure 6:**
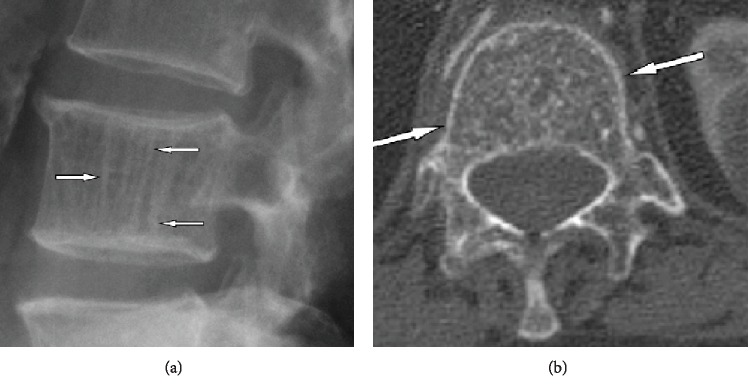
Lateral radiograph (a) exemplifying typical vertical striations due to thickened trabeculae. Axial CT scan (b) shows distinguishing polka-dot sign. Reprinted from [16].

The clinical course of neurological symptoms as caused by vertebral body hemangiomas tends to be slowly progressive over weeks to months. In this case, our patient progressed from having normal neurology to frank myelopathy with severe gait ataxia in less than 7 days. The rapidity of presentation of myelopathic symptoms is typical for cord compression from infection or rapidly growing metastatic disease. Rarely is such rapidly progressive myelopathy the result of an aggressive VH and requires a high index of suspicion for diagnosis. The level of our patient's lesion was at T1, which is atypical. Although the thoracic spine tends to have the highest propensity for vertebral hemangiomas with extraosseous extension, with 90% of lesions being in this part of the spine, approximately 75% of them occur between T3 and T9 [10]. Histologically, vertebral hemangiomas consist of vascular spaces lined with endothelial cells and thin-walled blood vessels. The vessels are surrounded by a fatty matrix and vertically oriented bone trabeculae. This gives the hemangioma its classic appearance on radiography or CT of parallel striations on sagittal views and the polka dot on axial views (Figure 6). However, the aggressive form of VHs is more likely to have an increased vascular component and less fatty content which contributes to its difficult differentiation from metastatic disease or primary bone malignancies based on imaging. They have a similar appearance to that of malignant tumors on routine STIR and T1- and T2-weighted MRI images [16]. Some have suggested using dynamic contrast-enhanced MRI perfusion to allow for differentiation between metastatic disease and aggressive VHs by gleaning information about the microvascular environment of the lesion [17].

Potential treatments after diagnosing an aggressive vertebral hemangioma include radiation therapy, endovascular embolization, vertebroplasty, ethanol injection, and surgical intervention. Although we were unable to make a diagnosis using diagnostic imaging studies prior to surgery, given the rapid progression of myelopathy and severe spinal cord compression, surgical intervention was elected for treatment. Given the unknown diagnosis at the time of presentation and the rapidity of his neurological decline, we opted to proceed posteriorly first to stabilize the spine with instrumentation and perform a laminectomy to decompress the spinal cord. Once the pathology returned with the diagnosis of aggressive but benign VH, we then performed the anterior corpectomy preceded by preoperative embolization to minimize blood loss.

The use of preoperative embolization is still controversial as it related to aggressive VHs. This is due to the most common areas for aggressive VH being in the midthoracic spine, where there is a potential watershed area for spinal cord blood supply. In these cases, embolization may be risky and should be deferred if there is one dominant feeder to the spinal arteries. Corpectomy with cage reconstruction was chosen based on the degree of anterior vertebral body involvement. Postoperatively, we delayed radiation by 3 months to allow for the fusion biology to be well underway and then opted to deliver 40 Gy of radiation to the surgical area to reduce the chances of recurrent disease which has been recommended by previous studies [18]. Postoperative radiation therapy is controversial as well. In this case, an inaccessible tumor was left in place and radiation has been shown to be preventative of tumor recurrence under these circumstances [3].

## 4. Conclusion

Rapidly progressive myelopathy due to an upper thoracic aggressive VH is rare but must be included in the differential diagnosis of these lesions. CT findings of bony expansion and trabecular bone striation should raise the index of suspicion for this type of lesion. In the face of rapidly progressive neurological decline, the treatment of choice is surgical intervention to decompress the neurological elements and resect as much of the tumor as possible. Preoperative embolization and postoperative radiation therapy can be useful adjuncts in the treatment of these tumors.
